# The Book World of Medicine and Science

**Published:** 1907-01-12

**Authors:** 


### THE BOOK WORLD OF MEDICINE AND SCIENCE.

DENTAL MATERIA MEDICA THERAPEUTICS AND PRESCRIPTION WRITING. By ELI H. LONG, M.D., Dental Professor, University of Buffalo. Illustrated with 7 engravings and 18 coloured diagrams. Second edition, thoroughly revised and enlarged. (London: Hodder and Stoughton.)

THIS work is dedicated to the memory of Dr. W. T. G. Morton, who first made known anæsthesia by ether. It is an attempt to classify into groups, and to give the action and uses of, therapeutic agents most likely to be useful to the dentist. The aim has been to cover what is essential and at the same time to supply in a concise form a broader view of the science of pharmacology. The work deals especially with the remedies and appliances which belong properly to dental medicine, forming a well-condensed text-book of therapeutics covering all the ordinary demands of the general practitioner and of the dentist. It deals with most of the subjects found in an ordinary work of general pharmacology, such as the constituents and preparations of drugs and their administration, as well as their mode of action. Depletives, counter-irritants, escharotics (under which the mineral and organic acids are treated of), the caustic alkalies, phenol, the silver salts, arsenious acid, are substances which receive special note under the heading of local remedies. We note in passing that the author precludes the use of iodoform in dentistry on account of its odour, but we have been unable to find a substitute that fulfils the same purpose in root-filling and with such lasting results. The uses of arsenious acid (arsenii trioxidum) in dentistry are tabulated very concisely, and the whole chapter is of special interest. After dealing with astringents and hæmostatics the work provides an important chapter on the cleansing agents, under the term detergents, and points out the adaptation of the alkalies and milder antiseptics to the cleansing of the mouth, teeth, throat, and nasal chambers, and of the stronger to the cleansing of foul ulcers, putrescent pulp canals, and such like matters. The chapter on antiseptics is particularly useful, and the paragraph on sterilisation should prove to be valuable to those practitioners who had not the benefit of practical training in aseptic work which the younger members of the profession are now acquiring at the various schools. Under the heading of local analgesics he mentions the various preparations employed to paralyse the sensory nerve-endings, and gives observations as to the dosage and mode of use of each. He illustrates the action of drugs by coloured diagrams which play a useful part in demonstrating the effect of the various anæsthetics on brain, spinal cord, heart, and the vascular and muscular systems. The whole work is rendered complete as a reference book by a very remarkable general index. The book is beautifully printed and thoroughly well illustrated. It is a safe guide to intricate problems which continually beset not only the student and dentist, but also the general practitioner of medicine. It will, moreover, be sure to find a place among the text-books of the dental student, and will be a valuable addition to those now in use at the different dental hospitals.

THE CLIMATE OF LISBON. By Dr. D. G. DALGADO. (London: H. K. Lewis. Pp. 50. 2s. 6d.)

AFTER the Peninsular War there was a period when Portugal enjoyed a well-earned reputation as a winter residence for invalids, and if it lost much of this reputation later, this was simply because many other places offered superior advantages, both in the accommodation provided for travellers and also in the greater ease with which these other resorts could be reached from England. Much has recently been done to remove these drawbacks, and we shall be surprised if the Portuguese Riviera does not recover much of the favour it at one time enjoyed. The pamphlet under notice shows quite clearly how substantial are the claims now made for this delightful stretch of country; and Dr. Dalgado points out that the winter temperature of Mont' Estoril on the Bay of Cascaes is higher than that of several of the most frequented of the Mediterranean health resorts. For instance, during December, January, and February the mean temperature at Mont' Estoril is respectively 10.56°, 10.21°, and 11.13° Centigrade; while for the same months Nice is 8.24°, 7.36°, and 8.12°; and Catania, near Taormina, is 11.3°, 10.1°, and 10.9°; and what is of equal, or greater, importance, the diurnal variations are less at Mont' Estoril than in either of the other places named. Frost and snow are almost, if not quite, unknown at Mont' Estoril, and it is much sheltered from the winds by the Serra of Cintra and by the low-lying "Montes" of Estoril. Nevertheless, as we remarked in a former notice on Portugal, it is clear that the country must experience some of the Atlantic gales. The drainage at Mont' Estoril is said to be quite good, the water supply excellent, the air very pure, and there are pleasant walks along the shore and in the woods. On the occasion of a visit we made early in March we found the chestnuts and other ornamental trees in blossom, and the roses were in full bloom. There are two good hotels at Mont' Estoril, and we believe another is about to be built at Cascaes, which we think is a better site than Mont' Estoril. The Portuguese Riviera has one very great advantage over most of the other Continental health resorts: it is within forty minutes' railway journey of the capital of its country; and Lisbon is a very nice city to live near or to visit. One point cannot be overlooked, and it should have the immediate attention of the Spanish and Portuguese authorities. That is, a considerable acceleration of the train service from the French frontier, so that those invalids who dread the sea might travel overland in reasonable comfort. At present the journey entails upwards of sixty hours of direct travelling. From Southampton, Lisbon may be reached by the Royal Mail Line and from London by the Hall Line, or by the Booth Line from Liverpool or Havre. The latter company offers very favourable terms to its passengers. Much information concerning Portugal may be derived from Dr. Dalgado's pamphlet, and intending visitors would do well to provide themselves with copies; but, we think it a pity that it could not be sold for less than two shillings and sixpence. Invalids should bear in mind that consumptives are not admitted to Portuguese hotels or boarding-houses, as consumption is looked upon as an infectious disease.

